# Advanced Optogenetic-Based Biosensing and Related Biomaterials

**DOI:** 10.3390/ma14154151

**Published:** 2021-07-26

**Authors:** Mihaela Gheorghiu, Cristina Polonschii, Octavian Popescu, Eugen Gheorghiu

**Affiliations:** 1 International Centre of Biodynamics, 1B Intrarea Portocalelor, 060101 Bucharest, Romania; cpolonschii@biodyn.ro; 2 Molecular Biology Center, Institute for Interdisciplinary Research in Bio-Nano-Sciences, Babes-Bolyai-University, 400084 Cluj-Napoca, Romania; opopescu.ubbcluj@gmail.com; 3 Institute of Biology Bucharest, Romanian Academy, 296 Splaiul Independentei, 060031 Bucharest, Romania

**Keywords:** optogenetic (light-responsive) biomaterials, cell-based biosensors, time-lapse multiparametric assays, optogenetic control, cell dynamics

## Abstract

The ability to stimulate mammalian cells with light, brought along by optogenetic control, has significantly broadened our understanding of electrically excitable tissues. Backed by advanced (bio)materials, it has recently paved the way towards novel biosensing concepts supporting bio-analytics applications transversal to the main biomedical stream. The advancements concerning enabling biomaterials and related novel biosensing concepts involving optogenetics are reviewed with particular focus on the use of engineered cells for cell-based sensing platforms and the available toolbox (from mere actuators and reporters to novel multifunctional opto-chemogenetic tools) for optogenetic-enabled real-time cellular diagnostics and biosensor development. The key advantages of these modified cell-based biosensors concern both significantly faster (minutes instead of hours) and higher sensitivity detection of low concentrations of bioactive/toxic analytes (below the threshold concentrations in classical cellular sensors) as well as improved standardization as warranted by unified analytic platforms. These novel multimodal functional electro-optical label-free assays are reviewed among the key elements for optogenetic-based biosensing standardization. This focused review is a potential guide for materials researchers interested in biosensing based on light-responsive biomaterials and related analytic tools.

## 1. Use of Engineered Cells for Cell-Based Sensing Platforms

Applications of live cells in the field of (bio)materials and biosensors range from building blocks for tissue engineering [1,2] to bioreceptors [3,4] and transducers in sensing configurations [4]. The latter dual capacity explains the sustained progress in the ‘cell-based biosensors’ [4] research field. As natural sensors, cells continuously process information from their environment and yield complex responses (e.g., by migrating, expressing genes, relaying signals to other cells, differentiating, growing or dying) with biosensing relevance by quantitative appraisal of cellular responses as a function of impending stimuli. Indeed, exposure to analytes (often noxious) prompts deviations of key cell parameters(e.g., membrane potential, cell volume, electrolyte concentrations, pH, reactive oxygen species—ROS) from their physiological relevant ranges, modulates specific cellular responses (changes in state or activity of cells in terms of, e.g., morphology, gene expression, enzyme production) and triggers homeostasis-restoring mechanisms. Failure of these mechanisms leads to cell cycle alterations and eventually to cell death, giving cell-based platforms their unique ability to reflect physiologically relevant functional information concerning stimulus propensity/efficacy to elicit bio-effects, and not merely the analyte’s concentration or binding. Applications of sensing platforms based on live cell analysis [2,3,5,6,7,8,9,10,11,12] are diverse, ranging from investigation of basic cellular functions in relation to aging, disease pathogenesis and pathology progress to assessment and monitoring of drug-ligand interactions and the effects of bio-available/active agents. In relation to adaptability to high throughput-screening (as also demonstrated in conjunction with reporter gene technology [13,14,15,16,17,18]), cell-based platforms enable the in vitro investigation of ADMET (absorption, distribution, metabolism, elimination and toxicity) properties well in advance of animal studies.

Implementation of the cellular-based sensing platforms raises important challenges. The first and foremost is the typically long associated response/analysis time, which spans from hours to days, in particular for assays based on signal-transduction events that occur downstream of receptor activation and require gene expression (challenge 1). The possibility of interference from other intracellular pathways (challenge 2), limited specificity (challenge 3) as well as the high variability of cell response (challenge 4) and sensitivity to cell environment (e.g., growth conditions)—challenge 5, relate to additional weaknesses encountered in cell-based platform use. To improve robustness (i.e., to separate the specific signal from nonspecific interferences), the use of a reference signal (either internal or external) to normalize the analytical response is often a prerequisite, thus adding complexity to the biosensing platform configurations (challenge 6). Furthermore, there is high variability in both the type of reporter cells (challenge 7) as well as in the analytical tools used for the quantitative appraisal of cellular responses (challenge 8), hindering standardization.

Optogenetics [19] is the combination of genetic and optical methods to achieve targeted gain or loss of function of (spatio-temporally) well-defined events in specific cells or living systems. This active and sensitive control, via light, of cell’s intrinsic dynamic stability, known to play the central role in shaping the response of cells to external perturbation, be it toxic or stimulatory, is key for innovative designs of smart biosensing concepts. Moreover, it provides a wide panoply of synthetic tools for the design of engineered cell reporters with fast modulation of cellular reactivity [5,6], improved specificity and more characteristic responses, compatible with time-lapse cell-based assays, thus holds the potential to eliminate such bottlenecks and challenges of the classical cell-based biosensing assays and to reach utmost relevance for this particular biosensing field [4].

In this review, we address the emerging optogenetic applications to the field of cell-based biosensing and cellular reporters. We bring into focus very recent approaches supporting optogenetics use for the development of enabling biomaterials and new biosensing concepts for highly sensitive live cell-based bio-sensing.

Given the availability of extensive recent literature (reviews included) and annotated databases, we outline the current suite of optogenetic tools at hand to the researchers in the field of (bio)engineered cellular materials that could be particularly relevant particularly since the emerging applications of this interdisciplinary and highly dynamic field are somewhat scattered and emergent, despite their exceptional potential impact. We also call attention to studies that increase our understanding of and improve our ability to engineer optogenetic-based biosensing materials and analytic concepts.

This focused review targets the key elements of an ideal cell-based biosensing platform, i.e., the engineered cell reporters, the toolbox available to achieve control of cellular dynamics and the suitable analytics technologies for improved biosensing relevance.

Based on the current knowledge, it is feasible to envision that upon smart integration and advanced material’s design, optogenetically powered cell-based biosensors will soon evolve from being mere tools for detection of specific analytes towards highly sensitive platforms of bioeffects of various, including unexpected stimuli/stressors in their medium. The novel cellular biosensing platforms hold promise as multiparametric devices for real-time monitoring and assessment [4,20] towards theranostic tools, offering unprecedented diagnose and treatment options and unique personalization avenues [6].

## 2. Optogenetics in Sensing

The utility of using light to perturb and to monitor biological processes is not a new concept [21], yet the emergence of optogenetics can be pinned down in 2005 when the microbial opsin genes were first demonstrated as control tools in neuroscience to safely confer cells’ light-detection capability and defined effector function. Indeed, owing to their structural simplicity (both light-sensing and effector domains are encoded within a single gene) and fast kinetics, microbial opsins provided the first precise and modular photosensitization components for introduction into non-light-sensitive cells to enable rapid optical control of specific cellular processes [2,19,22].

Ever since, obtaining compatible readouts and performing analysis, such as targeted imaging or electrical recording of evoked activity, became light-enabled. Originally developed to regulate neuronal excitability, optogenetics is increasingly used to control other cellular processes; sensing, actuation and wide control of bio functionalities of genetically modified cells were demonstrated with unprecedented spatiotemporal resolution and parallelism.

Not surprisingly, the last 15 years witnessed a ground-breaking impetus of optogenetic applications and tools (for applications in cardiac research see [23], one of the many recent reviews in the neuro and cardiac fields) progressing from basic research to (currently prospective) clinical therapeutic applications and industrial efforts to employ optogenetic strategy toward disease interventions [24]. In contrast, the biosensing applications of light-responsive cellular platforms are at an early developmental stage.

Recently [25,26], we demonstrated novel cellular sensing platform concepts based on optogenetic modulation of cell homeostasis, capable of meeting many of the challenges faced by cell-based biosensing platforms. Light-enabled actuation was demonstrated to provide increased cell reactivity and enhanced cellular responses to even minute exogenous stimuli, as well as inner reference together with the capacity to discriminate between different types of analytes and analyte concentrations. While using only one model tool (i.e., Channelrhodopsin 2 and 470 nm LED illumination) in the vast available optogenetic toolbox [27,28] in conjunction with electrical and optical assays, these studies set the grounds for advanced biosensing concepts that enable quantitative, real-time, label-free assessment of model cell dynamics in respect to different levels of homeostasis perturbation and demonstrate that optogenetic engineered cells enable the advancement of powerful approaches in cellular platform development for bioanalytics.

**Scheme 1:**
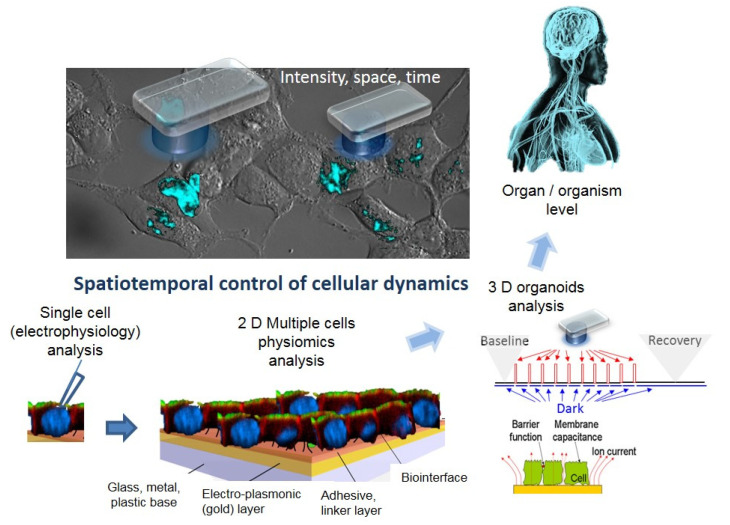
An overview of membrane-acting optogenetic control, and compatible phenotyping readouts across relevant cell-based biosensing platforms, from single cells to 3D tissue mimics (organoids), to control and assess the dynamics of cellular responses.

The spatiotemporal modulation of cell dynamics, bestowed by membrane acting optogenetic control, according to Scheme 1, was applied on single cells and 2D cell monolayers in conjunction with optimized light–dark cycles and electrical and optical analyses to assess cellular dynamics characteristics and their stimuli induced changes. The necessary progress towards 3D cultures and organisms (Scheme 1) is still challenging.

Such platforms [25] integrate many of the key elements of successful cellular sensing tools using light as a gear that are highlighted in the following subsection.

### 2.1. Key Elements Involved in Cell-Based Biosensing

(a) Active and sensitive control, via light, of cell’s intrinsic dynamic stability, known to play the central role in shaping the response of cells to external perturbation, be it toxic or stimulatory. The selective cell stimulation can be achieved across wide cellular scales and can be combined with electrophysiology or electro-optical assays of cellular status for cell-based sensing platforms, according to Scheme 1. Laser light of specific wavelengths can be used to achieve cellular control spatially restricted to single cells/subcellular volumes, whereas in larger 2D cell sheets, light-emitting diode, LED-based optogenetic stimulation can provide electrical pacing integrable with time-based impedance assays.
(b) Minimally perturbing actuators (as provided by optogenetics toolbox) compatible with live reporters. The reporter cells, i.e., live cells modified with light-sensitive molecules, are the key components of a specialized class of biosensing platforms that highlight the response of cells toward quantitative evaluation of the changes in their microenvironment, including occurrence/presence of bioactive molecules. There is a wide variety of robust excitable/non-excitable cells with tailored light responsiveness and homeostatic control that can be developed and complementary tested (e.g., via electrophysiological tools) and interfaced with electro-analytic assays with integrated controlled microfluidics and optical stimulation.
(c) Fast and affordable electro/optical-analytics amenable for standardization. Indeed, optical methods can be easily implemented and are relatively inexpensive in particular when combined with multiparametric optical readouts of cell physiology within microscopy platforms [29]. Moreover, time-lapse fast impedance assays [25,26] on electrode adherent cells are capable of assessing with exquisite sensitivity the minute changes of cellular state triggered by exposure at bioactive compounds/stimuli and reveal, alone or in combination with optical assays [30,31,32], fast and affordable detection avenues and provide ideal transducers/cell physiomics analysis platforms.

### 2.2. Advantages and Disadvantages of Cell-Based Biosensors and Optogenetic Approaches

Cellular optogenetics offers a modular and generalizable set of tools for controlling specific biochemical reactions in space and time and control of the properties of biological systems. This control achieved via specific actuators can accelerate the progress of cellular responses to certain stimuli (addressing challenge 1 of standard cellular platforms). It can also provide inner reference dynamics eliminating the variability of cellular responses and the need for control channels/cultures. The ever-expanding portfolio of optogenetic actuators and sensors, the ease of use, the possibility to control illumination strength, tune the wavelength for cross-scale applications (e.g., used red light for improved tissue accessibility) and to create and project patterns of optical stimulation are the main advantages of optogenetic approaches, further recommending optogenetics for implementation in cell-based biosensing platforms.

Using optogenetic inputs, it is also possible to achieve (a) the maximum transcriptional output of selected intermediate genes; (b) control of cell fate decision making (to either differentiate or to maintain a multipotent progenitor state); (c) control over the dissolution/aggregation of phase-separated protein clusters relevant to establish light-defined spatial distributions.

Moreover, optogenetics eliminates the main disadvantage of the photo-responsive chemically caged inducers related to the necessity to either apply the caged molecules exogenously or to tediously introduce caged amino acids into proteins.

Optogenetics is clearly not an ideal land. The main challenges relate to: (1) the risk to alter the cell physiology when engineering cells, beyond simply adding light sensitivity, (2) the possible detrimental cell responses caused by intense light stimulation needed to activate the light-responsive molecules and (3) the thermal and electrical (such as photovoltaic) confounding effects. Since grossly addressed by individual component developments (whether at cell level or at stimulation/analytic levels), presenting the progress in alleviating these disadvantages exceeds the focus of this review.

### 2.3. Envisaged Optogenetics Applications to the Field of Cell-Based Biosensing/Cellular Reporters

As supported by the advantages mentioned in Section 2.2, the optogenetics-powered, cell-based biosensing and cellular reporters could have an exceptional potential impact and wide addressability in the following fields:

(a) Point of Need (PON) quantitative analysis of various stressors’ bio-impact;
(b) Assessment of disease phenotypes and pharmacological modulation for preclinical assays and pharmaceutic industry;
(c) Ingestible/implantable bioelectronic biosensing devices and theranostic platforms;
(d) Development of smart cell sentinels.

While the list is not exhaustive, as indicated in Table 1 and detailed in the following sections (in particular Section 6), many of the applicative fields were addressed (merely) at the proof of concept level with some exciting results, while others are yet to emerge. Such is the field of 3D tissue mimics that can be used to evaluate pharmacological profiles as proxies for organ and organism level control that lag far behind reported applications based on 2D/3D cellular biosensing platforms, the focus of this review.

## 3. Generation of Optogenetically Modified Cell Lines and the Complementary Tools for Their Characterization

One key element in the development of cell-based sensing platforms is the nature/type of reporter cell. In this respect, optogenetics has high flexibility. Although it was originally applied in neural tissues to manipulate neuronal activity, the technique is widely applicable for other types of cells (Table 2).

Application of optogenetic tools greatly exceeds neurons and cardiac cells, as demonstrated through reports on stem cells [33], connective tissue cells [49], plant cells [50], yeasts [51,52] and bacteria [53] that are starting to amass supported by both transient and transgenic formats.

As implemented by us [25,26], optogenetically modified cell lines (e.g., ChR2-expressing cells) can be easily generated by means of site-specific recombination (for instance, using the HEK293-Flp-In System—Life Technologies, Invitrogen). HEK293 Flp-In cells contain a genomic targeting site (e.g. flippase recognition target FRT) for the reproducible insertion of single copies of cDNAs. Prior to transfection, achieved, for instance, via electroporation, the desired cDNAs need to be first cloned into a dedicated vector (pcDNA5/FRT). Selection media cultivation results in the generation of clones carrying and expressing the desired cDNAs for a ChR2-YFP fusion protein and other ion channels, at will (e.g., cDNA for ROMK1 [25,26], a 2A peptide adaptor sequence and ChR2-YFP). 

Expressing more than one photosensitive protein in target cells could also be advantageous. Different photosensitive proteins can produce different electrophysiological effects, enabling researchers to bidirectionally and reversibly manipulate biological functions, including membrane voltage and cellular responses.

However, in the design and implementation of optogenetically modified cells, one should be aware that optical actuation methods have their own limitations presented in Section 2.2 which need to be considered. First, while ChR2s are generally well tolerated, additional ion channels (either regular [25,26] or photosensitive) may alter the physiology of the cells beyond simply adding light sensitivity. Second, intense light can cause detrimental cell responses. While this has been traditionally considered to be of no concern in most optogenetic experiments, researchers should ensure that wavelengths and optical power effects are well characterized and that actuation involves optimal reversibility. Third, ChR2 transgene expression within a sample can be variable. Since photocurrent depends on channel number and type, cells may display heterogeneous responses to the same light intensity. This becomes increasingly relevant as cell number reduces towards single-cell experiments or as per high-throughput approaches. 

**Figure 1:**
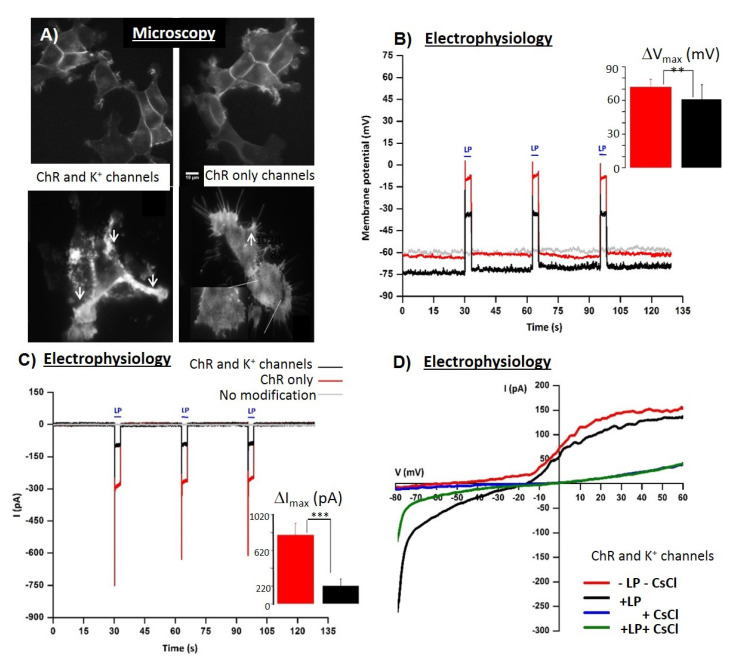
Complementary characterization of the functional expression of genetically encoded ion channels (the ChR2 and ROMK1 channels). **Microscopy** (**A**): different levels of ChR2-YFP expression can be quantified using fluorescence imaging (fluorescence images, scale bar 10 µm) as well as their membrane distribution using Total internal Fluorescence Microscopy (lower panel scale bar 1 µm). **Electrophysiology**: (**B**) Current clamp data reveal the (resting) membrane potential values (V*m*) and their changes upon illumination as a function of channel repertoire; inset membrane potential values changes (ΔV*max* = V*max* (pulse) − V*m*) upon illumination—enabling comparison between light-induced depolarization changes (significance level ** *p* < 0.01, *n* =5) for ChR-only expression and ChR and K^+^ channels, respectively. (**C**) The Voltage clamp data reveal transmembrane currents (I) upon illumination and enable quantification of functional channel density. Inset, the amplitude (ΔI*max* = I*_0_*− I*max* (pulse)) of the current elicited by the first LP is significantly (*** *p* < 0.001, *n* = 5) higher for cells expressing ChR only, corresponding to the functional ChR2 channels opening. For cells with a more complex channel repertoire (e.g., cells expressing both ChR and K^+^ channels), separating the individual transmembrane current components require specific channel inhibitors (see D). (**D**) Representative I-V plots corresponding to −80 mV ÷ +60 mV voltage ramps with K^+^ channel inhibitor CsCl and on–off illumination (green/blue curves) demonstrating functional, tandem expression of K^+^ channels and ChR2 channels (as controls we use −LP-CsCl as well as +LP-CsCl current values, i.e., red and black tracks, respectively).

Optical and electrophysiological tools (see Figure 1) should thus be used to characterize the engineered cells, i.e., the quality of the genetic transformation as a prerequisite step in optogenetic cellular platform development. Especially voltage and current clamp assays (Figure 1, Electrophysiology B-C) are standard yet powerful assays for characterization of the functional expression of genetically encoded ion channels and the light control effectiveness at the single-cell level. For cells expressing more than one photosensitive/active protein, specific electrophysiological tools are available for characterization upon implementation of specific channel inhibitors and voltage ramps protocols (Figure 1, Electrophysiology D).

**Scheme 2:**
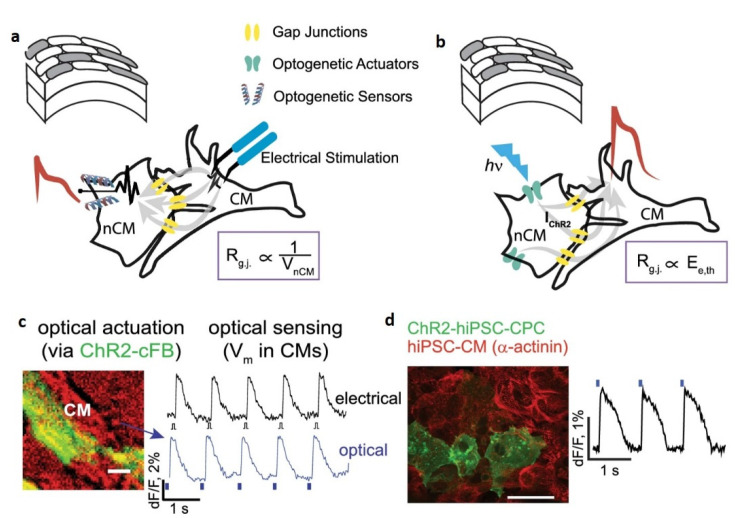
Optogenetic methods offer new ways for assessing hetero-cellular coupling as either (**a**) ‘optogenetic–sensor’ variant, where coupling is typically confirmed by measuring membrane potential fluctuations (VnCM) in non-cardiomyocytes (nCMs) cells expressing genetically encoded voltage indicator (GEVI)/ genetically encoded calcium indicator (GECI) and connected to cardiomyocytes CMs undergoing excitation or (**b**) ‘optogenetic–actuator’ approach where coupling can be quantified by the light needed to trigger excitation in the CMs via the light-sensitive non-cardiomyocyte nCMs type cells, i.e., E_e,th_. This ‘optogenetic–actuator’ approach is the recommended way to implement the tool for both ChR2-cFB and CMs (**c**) as well as for light-sensitized human cardiac progenitor cells (ChR2-iPS-CPC) and non-transformed human induced pluripotent stem cells derived CMs (hiPSC-CMs) (**d**). Redrawn from [54].

For certain biosensing applications and for difficult to transfect cells in native tissue settings, light-responsive formats can be implemented without the direct optogenetic modification of the reporter cell. One such example is the OptoGap [54] format in which a contactless assay for the electrical coupling strength was demonstrated based on selective illumination of non-excitable cells (robust and well-characterized) that express optogenetic actuators (e.g., a light-sensitive protein acting on membrane voltage) and the optical/electrophysiological sensing of induced response to (excitable) reporter cells that are light-insensitive, as modulated by coupling strength, according to Scheme 2. As such, optogenetics-inspired methods can provide quantitative means to assess cell–cell coupling in both 2D multicellular settings, as well as hetero-cellular coupling in the native tissue setting. Consequently, the ‘tandem cell unit’ originally developed by electrically coupling cardiomyocytes with non-excitable HEK293 cells that express an optogenetic actuator was implemented with a two-layer patterned co-culture of fibroblasts i.e. non-cardiomyocytes cells (nCM–ChR2FB) and cardiomyocytes (CM) as a test platform for a direct method for appraising the coupling in multicellular tissues, according to Scheme 2.

**Scheme 3:**
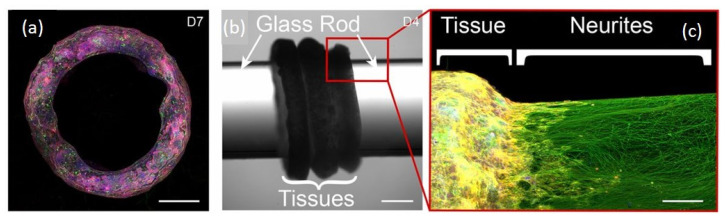
(**a**) Confocal of compacted toroid neural tissue mimic (NTM). (Scale bar: 1 mm.) (**b**) Installation of toroid NTMs (Day 4 from seeding) on functionalized glass cylinders after compaction in the 3D-printed mold. (**c**) LightSheet image of neurites extending throughout the glass rod (β-TubulinIII, green; F-actin, yellow) after 3 days. (Scale bar: 1 mm; 500 μm.) Redrawn from ([37], PNAS 2019].

Recent improvements in three-dimensional (3D) cell culture have improved the physiological relevance of cell-based assays because they mimic cell growth in vivo and preserve cell viability, pathway activity and global gene expression profiles, leading to increased predictive power [37]. Meanwhile, as widely demonstrated in 2D cell cultures and in vivo settings for neuronal, cardiac and gastric models, 3D optogenetic mimics are only recently reported [37]. In their work, Pagan-Diaz et al. present an approach to form functional in vitro neural tissue mimic of different shapes (cuboid, rod, toroid) using stem cells, a fibrin matrix and 3D-printed molds. With optimized cell-seeding protocols, characterized internal structure and remodeled extracellular matrix and thorough validation of the electrophysiological activity using optogenetic cells (mouse embryonic stem cells modified with ChR2H134R), the construct (in particular the toroidal shape) is an interesting platform for 3D optogenetic platform development. We consider that the possibility to interface with a glass rod (see Scheme 3) could provide an added bonus if the latter is replaced by moldable hydrogels with optogenetic functionality (i.e., light-guiding hydrogels or with light-modulated properties).

## 4. The Optogenetic Toolbox

The optogenetics toolbox is a generical term covering the wide spectrum of light-sensing components with distinct spectral properties that enable versatile control of cellular functions and modulation of cell homeostasis with light. 

The membrane potential is in itself the main cellular homeostasis regulator—acting as a liaison between the extracellular and intracellular milieus, as well as a powerful cellular actuator and sensor. As actuator, membrane potential modulations have exquisite roles in signal transduction and propagation, in activation of intracellular processes (e.g., by regulating G-protein cell receptors (GPCRs) family of membrane proteins that translate the binding information from a broad spectrum of extracellular ligands into a range of signaling pathways in the cell), in cell-to-cell communication, in the control of cellular functions (e.g., contraction, release of insulin) hence strongly shaping cell–environment integration. As a sensor, the membrane potential dynamics reflect the cellular status and provides relevant information for establishing the physiological, pharmacological role for specific analytes (drugs, chemicals or nanoparticles) [34,61,62,63], as demonstrated by approaches with microelectrode array (MEA) assays. 

**Figure 2:**
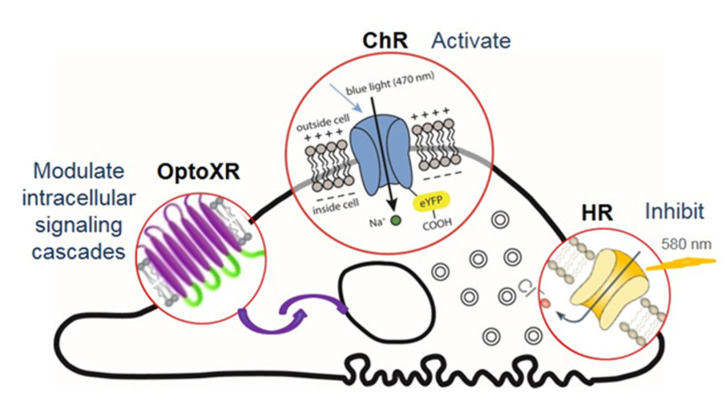
Common light-sensitive proteins in optogenetics research: channelrhodopsin ChR allows inward photocurrent of non-selective cations (Na^+^ > K^+^ >> H^+^ >> Ca^2+^), which can evoke cell depolarization under 470 nm wavelength light (with activating effect for excitable cells). Halorhodopsin HR, an anion (Cl^−^) channel/pump, can produce membrane hyperpolarization and electrophysiological inhibition under 580 nm wavelength light. OptoXR (a G-protein-coupled receptor) is used to modulate intracellular signaling cascades.

Central to optogenetic control of the membrane potential dynamics and cellular functions is the light-induced morpho-functional modification of photosensitive proteins, which usually function as ion channels, pumps or receptors (Figure 2). When delivered to target cells, the photosensitive proteins respond to light pulses of specific wavelengths changing the electrochemical gradient and the intracellular ionic equilibria and trigger transmembrane potential modulation or, as for G-protein-coupled receptors (OptoXR), modulate intracellular signaling cascades.

The optogenetic toolbox (Table 3) is vast and comprises light-sensing components (light-controlled ion channels and light-gated protein tools for ‘cellular optogenetics’ [57]) with distinct spectral properties that can be harnessed to engineer novel optogenetic means enabling precise control of biological functions in mammalian cells, with superior spatiotemporal resolution and addressability [64].

The building blocks are the opsins, the naturally occurring light-sensitive transmembrane proteins that are found in a variety of organisms ranging from microbes to primates.

The natural opsins are implicated in their host organisms in a variety of functions, including navigation towards sources of energy and away from hazardous environments (this implicates as well control of the beating of flagella) besides the ‘standard’ control of the intracellular concentrations of ions. In animal cells, opsins are implicated in vision and for modulating circadian rhythms.

Bacterial opsins were used in the first optogenetics experiments to control neuronal function and remain a primary source for new natural and engineered opsins. Engineering is meant to optimize their function (e.g., in terms of wavelength dependence, temporal response) and applicability. One such example is the development of Opsin/G protein-coupled receptor chimeric molecules, OptoXR, that merge an extracellular light-sensitive part and an intracellular G protein-coupled receptor (GPCR) to initiate a signaling cascade upon activation. With OptoXR type photosensitive proteins, the optogenetic toolbox reached new dimensions enabling more nuanced changes in cell activity than for mere bacterial opsins. This complex control is supported by (a) the variety of ligands that bind and activate these receptors that include besides light-sensitive compounds, odors, pheromones, hormones and neurotransmitters, as well as (b) the key role of GPCR in two principal signal transduction pathways relevant for cell physiology: the cAMP and phosphatidylinositol signal pathways.

In addition, optogenetics straightforwardly benefits from reporter gene technology that has unquestionably played a major role in the development of cell-based assays in the past decade [12,15,18,19,20]. As such, the toolbox of relevance for biosensing comprises both light-activated tools (actuators, from proteins, to DNA) as well as genetically encoded reporters (sensors) [58] that will be briefly presented below, in particular in relation to the possible biosensing applications.

### 4.1. The Optogenetic Actuators

Photo-responsive actuators gained a prominent role as gears of molecular activity [25] control, design components of new biosensing concepts and powerful biotechnological toolkit components in living animals, as well as in unicellular systems (yeasts [51,52], cellular reporters [54]/sentinels).

The optogenetic actuators are photosensitive proteins, which usually act as ion channels, pumps or receptors, that are delivered to target cells, where they respond to light pulses of specific wavelengths, evoke transient flows of transmembrane ion currents and induce signal transduction. They transform photon fluxes into transmembrane ion changes, thereby manipulating transmembrane potential on a millisecond scale to produce physiological reactions in mammalian cells and tissues in response to optical signals.

Optical actuators range from voltage actuators to biochemical actuators such as microbial rhodopsins, channelrhodopsins—ChRs, halorhodopsins—HR, archaerhodopsin-T (ArchT) and G protein-coupled rhodopsins (melanopsin).

Rhodopsins—two of the most common light-sensitive proteins in the arsenal of neurobiology are: channelrhodopsin 2 (ChR2) from the alga Chlamydomonas reinhardtii and halorhodopsin (HR) from the archaeon Natronomonas pharaonic [23]. These microbial rhodopsins, ChRs and NpHR, are common tools in optogenetics research that generate excitatory or inhibitory transmembrane currents to control the activity of excitable cells or generate transient membrane changes in a nonexcitable cell. 

ChR2 acts as an ion channel in the cell membrane. Upon irradiation with blue light (~470 nm), the conformation of the protein changes allowing cations (Na^+^ in particular) to pass through the channel pore. The resulting influx of Na^+^ depolarizes ChR2-expressing cells.

In contrast, NpHR acts as an ion pump moving Cl^−^ into the cell when illuminated with yellow light (~580 nm), determining membrane hyperpolarization.

With the development of molecular engineering, more variants of ChRs have been discovered or synthesized, such as CatCh [59], also enabling Ca^2+^ ion permeability.

Melanopsin [65] adds to the optogenetic toolbox an excitatory light-activated Gq-coupled receptor. Optogenetic control of cellular rhythms is enabled by the use of melanopsin that exists on the ganglion cells of the retina and is involved in the regulation of circadian rhythms. In a practical approach, optogenetic modulation of pacemaker activity of cardiomyocytes was demonstrated by Beyert [56] using melanopsin.

Importantly, the available repertoire of optogenetic systems based on genetically encoded light-sensitive proteins is more vast, comprising not only bacterial [23] and animal opsins [36] but also non-opsin-based optogenetic actuators [66]. The later include light-oxygen-voltage-sensing domain (LOV) [67], cryptochrome (CRY2), phytochrome (PhyB and BphP) and fluorescent protein (FP)-based photosensitive domains (Dronpa and PhoCl) [66].

Furthermore, exotic optogenetic actuators are also reported that include light-activated enzymes. Such an example is the photosensitive membrane protein, KillerRed [60] that acts as a photoinducer of reactive oxygen species to produce cytotoxicity and trigger cell degeneration and death and is fundamental for recent phototherapies [70,71].

### 4.2. The Optogenetic Reporters

Cell-based assays utilizing reporter gene technology [15,18,19,20] (e.g., green fluorescent protein, β-galactosidase, luciferases) have been widely exploited for biosensing, as they provide useful information about the bioavailability and cell toxicity of target analytes or can be used to monitor the cellular events associated with signal transduction and gene expression. In practical applications, genetically encoded biosensors [72] gained popularity by reporting on in situ enzymatic activities or the presence of a wide range of molecules such as ions, metabolites and messengers. The long assay time due to gene transcription and translation is one of the main drawbacks of reporter gene technology-based cell biosensors.

While spearheaded by tools to control membrane voltage, the more general concept of optogenetics includes the use of a variety of genetically encoded probes/reporters [73] for physiological parameters ranging from membrane voltage and calcium concentration to metabolism [58]. These more advanced genetically encoded biosensors combine the sensing unit that detects changes in the signal of interest and a fast-responding reporting unit that converts these changes into a quantitative readout. Notable, optogenetic fluorescent probes (FP) were demonstrated capable of breaking the long assay time limitation and were applied for optical imaging of/reporting on fast calcium and voltage dynamics in cardiac cells and tissues [74] with outstanding potential for biosensing.

On one side, the availability of calcium-dependent photoproteins enables the development of functional cell-based high throughput sensors (HTS) for intracellular calcium monitoring and, in particular, for the study of G protein-coupled receptors (GPCRs), which are the target of one-third of all marketed drugs [75]. Optogenetic Ca^2+^ indicator probes (red calcium indicator protein, R-GECO) enabled noninvasive phenotyping and drug testing in single cardiomyocytes or beta-cells [41]. On a more fundamental ground, optogenetic Ca^2+^ indicator probes are able to shed light on cell–cell communication [68].

On the other side, accurate measurement of the membrane voltage could elucidate subtle changes in cellular physiology. The temporal dynamics of microbial rhodopsin fluorescence [76] are able to report absolute membrane voltage [77] and shed light on other non-tractable bioelectric cell characteristics accompanying membrane voltage actuation. As an example, electrotonic coupling of excitable and non-excitable cells in the heart can be revealed by optogenetics reporters [78]. They offer cell-specific readouts or in vivo monitoring capacity of electrical activity [79] as demonstrated by OptoDyCE high-throughput, all-optical dynamic cardiac electrophysiology.

Moreover, optogenetic FPs were advanced from having exclusively sensing functions to also encompass optogenetic control of protein activity [80,81], protein clustering and signaling activity [55]. Traceable intracellular binding molecules provide new opportunities for real-time cellular diagnostics. Intrabodies, derived from antibody fragments of heavy-chain only antibodies of camelids (nanobodies), have emerged as highly versatile and attractive tools to control and visualize antigens within the context of living cells [69].

Different types of switchable systems were developed using light or small molecules as optogenetic or chemogenetic controllers/activators. In these types of systems, the transfer of biochemical information from the sensor domain to the actuator domain is mediated by conformational changes and aggregation processes that are able to be switched on and off. Optogenetic switchable intrabodies were developed by nanobody fusions with light-responsive proteins—opto-nanobodies (OptoNBs)—a class of chimeric photo-switchable proteins whose binding to proteins of interest can be enhanced or inhibited upon blue light illumination [82] and used for tunable light-induced modulation [83] (including depletion) of target proteins.

### 4.3. Optogenetic Control of Intracellular Signals

Optogenetic techniques for controlling intracellular signal transduction systems have been developed and applied in recent years by regulating intracellular signaling in a light-dependent manner [84] as well as achieving improved expression and effector function via combinations of opsins with membrane trafficking motifs [85]. Merging nanotechnology with optogenetic tools, worth noting are the genetically encoded toolkits of functionalized nanobodies for visualizing and manipulating intracellular signaling [86].

Optical tools that modulate protein-protein interactions emerged: opening the door to optogenetic control of kinases and transcription factors. The design of a tunable recognition unit and development of an aptamer-based near-infrared (NIR) light-responsive nanoplatform for manipulating the subcellular localization of specific proteins in their native states was reported [35].

The noninvasive and remote control of protein subcellular localization and inter-organellar contact sites are critical for cell signaling. Optical control of cell membrane phospholipids at membrane contact sites [85] provides additional means to real-time influence important cellular events, including membrane trafficking, calcium homeostasis and lipid metabolism, and overall light-controlled modulation of cellular reactivity [25,26] as well as appropriate insight on complex intracellular signaling pathways [87].

Optogenetic approaches were reported [88] for manipulating the localization and translation of specific mRNAs by trapping them in clusters. This clustering greatly amplified reporter signals, enabling endogenous RNA–protein interactions to be clearly visualized in single cells. Functionally, this sequestration reduced the ability of mRNAs to access ribosomes, markedly attenuating protein synthesis.

A spatio-temporally resolved analysis indicated that sequestration of endogenous beta-actin mRNA attenuated cell motility through the regulation of focal-adhesion dynamics. There is also a broad field of genetically encoded photo-responsive RNA nanodevices [89]. From mere light-regulated RNA switches based on the specific recognition of RNA aptamers towards a particular photo-induced isomerization state of a chromophore to actual photo-responsive RNA nanodevices engineered to specifically recognize a bacterial light-oxygen-voltage photoreceptor and sterically inhibit gene expression or generate reactive oxygen species (ROS) upon light irradiation and lead to cell structure damage and photodynamic therapy, these reversible and irreversible photo-regulated RNA nanodevices can be potentially used to precisely regulate cell functions. Optogenetics was combined with fluorescence resonance energy transfer, FRET, [90] to allow local microtubule manipulation and the visualization of trans-cellular contacts.

In summary, applying optogenetic tools allows controlling cell fate decisions such as proliferation and differentiation or to deliver therapeutic substances in a spatiotemporally controlled manner [91] down to cell organelle resolution [92]. Considered a new frontier 10 years ago [21], the delivery of optical control (whether biochemical or electrical) to well-defined subcellular domains or intracellular (e.g., membranous) compartments is now a reality.

### 4.4. (Upcoming) Engineered Opto-Chemogenetic Tools

With recent advances in designer probes, significant progress is expected from the combination of optogenetics and chemogenetics [93,94]. Chemogenetics is a method in which various types of protein classes are engineered to interact with small molecule chemical triggers. These engineered proteins are designer receptors, exclusively activated by designer drugs (DREADDs). DREADDS use a key-lock principle to selectively modulate cell activity by chemical means and are typically modified muscarinic G protein-coupled receptors (GPCRs). 

GPCRs recognize a wide variety of ligands, including odorants, photons, neurotransmitters, lipids, hormones, peptides and other small molecules, and regulate intracellular response to adapt to the changing environment. GPCR signaling can thus be regarded as a ‘universal’ modulator of patho-physiological responses. 

Accordingly, the combination of optogenetics and chemogenetics is ideally positioned to achieve multistage cellular control via ion channels activated by light (optogenetics) and G protein-coupled receptors (GPCRs) activated by drug-like small molecules (chemogenetics), and holds great promise to boost research in cell-based biosensing.

Important results to support this integration were recently reported. 

Novel fusion proteins, termed luminopsins, combine optogenetic probes with luciferase that emits biological light in the presence of a substrate. Both activatory and inhibitory tools can be developed that allow interrogation of cellular systems at different temporal and spatial resolutions by choosing either extrinsic physical or intrinsic biological light for their activation. Such expanded utility of luminopsins was demonstrated by Berglund et al. [95] by fusing luciferase with either channelrhodopsin to excite neurons (luminescent opsin, LMO) or a proton pump to inhibit neurons (inhibitory LMO, iLMO). As such, while preserving the ability to be activated by external light sources, LMOs expand the use of optogenetics by making the same opsins accessible to noninvasive, chemogenetic control. The rhythmic hippocampal circuit excitation over a large spatiotemporal scale was demonstrated, showing that the activity of neurons in vitro, ex vivo and in vivo could be controlled by biological light in addition to physical light [95]. 

Furthermore, luciferase-LOV bioluminescence resonace energy transfer, BRET, was demonstrated to enable versatile and specific transcriptional readout of cellular protein-protein interactions [96].

Moreover, chemi-optogenetic tools that enable protein recruitment with precise spatiotemporal control [97] to initiate a desired biological effect were demonstrated based on photoactivatable antibiotics towards advanced probing of cellular processes. The chemical versatility of photoremovable protecting groups combined with the biological specificity of self-labeling tags warrants a wide range of chemi-optogenetic tools that enable protein recruitment with precise spatiotemporal control ready to be explored.

### 4.5. Opto-Control of Cell Adhesion and Patterning for Improved Biosensing

Patterning of cells on electrodes can also improve the impedance characteristics of cell-based sensors since cell adhesion and spreading significantly affects [98] the impedance characteristics of cell-based sensors. As such, the work on new materials to control cell adhesion is of potentially high relevance. 

The progress in engineering dynamic biomaterials via the incorporation of photoreceptors from cyanobacteria or plants into polymer materials is reviewed by Horner et al. [38] with a focus on synthetic extracellular matrices with reversibly adjustable mechanical properties. One such material is phytochrome-based extracellular matrix (CyPhyGel), whose mechanical properties can be adjusted in a fully reversible, wavelength-specific (red domain) and dose-dependent manner with high spatiotemporal control.

Hopkins et al. [39] used a SNAP-tag® (New England Biolabs Inc, Ipswich, MA, USA) and its thiol-targeted substrate, benzylguanine-maleimide, to covalently attach blue-light-responsive proteins to collagen hydrogels. The resulting material (OptoGel) encompasses the native biological activity of collagen, stiffens upon exposure to blue light and softens in the dark. Interestingly, the light-guiding properties of hydrogels were demonstrated effective as early as 2013 when Choi et al. [40] reported cell-integrated polyethylene-glycol-based hydrogels for in vivo optical sensing and therapy applications. Using optogenetic, glucagon-like peptide-1 (GLP-1) secreting cells, light-controlled therapy and improved glucose homeostasis were demonstrated. Furthermore, real-time optical readout of encapsulated heat-shock-protein-coupled fluorescent reporter cells made it possible to measure the nanotoxicity of cadmium-based bare and shelled quantum dots (CdTe; CdSe/ZnS) in vivo. In conclusion, such biomaterials with light-guiding properties and cell adhesion control are important building blocks of new cell-based sensing platforms, with the added functionality of an enabling tool for the investigation of how cells respond to dynamic mechanical cues as occurring in living organisms or in next-generation bio-interfacing prosthetics.

## 5. (Label-Free) Analytical Tools Relevant for Optogenetic Cell Platforms

Label-free approaches have been widely used as a substitute for conventional reporter-based cell assays (based on labeling processes and end-point analyses) [4]. From the various label-free formats researched in the last few years, our group has been actively engaged in the development of dynamic formats based on cell-substrate impedance alone or in combination with electrochemical probes, nanoplasmonics and advanced microscopy. The successful implementation of optogenetic and chemogenetic techniques is expected to thrive thanks to ongoing advances in electrical assays (including implantable/flexible electronics), imaging microscopy and emerging multimodal technologies. Since the field is so vast that it exceeds the focus of this review, we will only briefly present some analytical tools in our portfolio [27,29,31,99,100,101,102] or related to them, amenable for optogenetic-based sensing concepts.

### 5.1. Electrical Impedance Sensing (EIS) Platforms

Electrical Impedance Sensing (EIS) platforms have gained an undisputed front place in the development of whole-cell biosensors due to their label-free monitoring capability of cell growth and proliferation, status of intercellular junctions [103], cell—substrate interaction, attachment, spreading, motility (including micromotion). 

Cell motility is ubiquitous among biological cells [104], allowing reversible change of interactions with other cells and with the extracellular matrix and requiring the coordinated activity of cytoskeletal, membrane and adhesion systems. Thus, quantitative measurement of cellular motility, interactions and response dynamics as offered by EIS is essential in various domains, from the study of the orchestrated immune response [105] to cancer diagnosis.

EIS is amenable to miniaturization, being based on the culturing of cells on an electrode surface and full integration with other complement technologies (surface plasmon resonance [31], micro fluidics and electrochemical probes [99]) and with controlled environments for improved versatility. For instance, an impedance-based method for real-time monitoring of cellular activity (cell adhesion, spreading and proliferation) was developed for quantitative assessment of specific carbonic anhydrase inhibitors’ effects on hypoxic cells [106]. EIS can provide information about cellular activity based on the measurement of induced capacitance or resistance changes occurring on various electrode configurations and is potentially ideal for the identification of new drugs [14]. Indeed, the real-time analysis based on impedance assay [106] allows discriminating between the inhibitory capacities of designer compounds and their inhibition mechanisms upon carbonic anhydrase IX expressed on live cells in monolayers and is effective for the screening and design of anticancer pharmacological agents. 

EIS is also amenable for high-throughput applications. Sensor arrays can be developed for real-time monitoring of cellular activity such as receptor-mediated endocytosis [107]. Interestingly, capacitance increased when adenoviruses or antibodies were bound to the receptors on the cell membrane, and decreased when the receptor–ligand complexes were internalized. Notable, as discussed in previous sections, the optogenetic tools are mostly membrane-based. Therefore, the biosensor concept reported by Mavrikou et al. [108] that is based on membrane-engineered mammalian cells bearing the human chimeric spike S1 antibody is potentially relevant. They demonstrated that the attachment of the protein to the membrane-bound antibodies resulted in a selective and considerable change in the cellular bioelectric properties measured by EIS.

**Figure 3:**
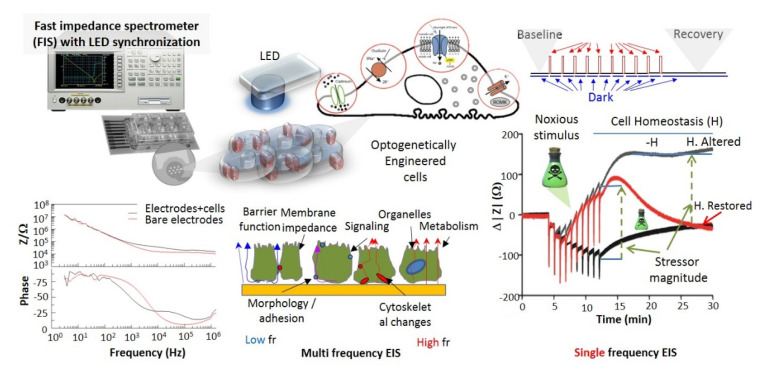
Schematic representation of a cell-based impedance sensing system with optogenetic control. The cells grown on the electrode act as insulators, impeding the flow of current, perturbing the impedance of the system as revealed by multi frequency Electrical Impedance Spectroscopy (EIS). Upon light control of optogenetic actuators, the characteristic impedance response (black) is rapidly and specifically modified upon addition of compounds able to alter individual cell characteristics (cell morphology, cytoskeleton, metabolism, cell signaling) or to disrupt the cell monolayer (affecting cell–cell communication or cell-surface attachment). Access to the whole dynamics, including the recovery phase, has predictive potential concerning the persistence of the compound-specific bio-effect (i.e., altered homeostasis/homeostasis restoration).

A new paradigm for impedance-based sensing was recently proposed by us [25,26]. It involves an optogenetically modulated cell-based sensing platform (according to Figure 3) that is suitable for in-field applications and capable of appraising the occurrence and the magnitude of cellular alterations induced by low levels of a bioactive/toxic compound on a short time scale and with exquisite sensitivity.

This novel biosensing platform incorporates HEK 293 transformed cells for light control of membrane potential, stably expressing ChR2 and K^+^ channels and electrical readout for multiplexed electro-optic) biosensing. The LED-based optogenetic control of otherwise non-electrogenic human cells was integrated into a noninvasive electro-optical analytical platform enabling quantitative assessment of the stimulus-dependent cellular response dynamics.

To test the functionality of the biosensing concept, we proved [25,26] that: (1) the light-induced perturbation of cell homeostasis is reflected in impedance assays through characteristic dynamics, quantitatively associated with light stimulus parameters (magnitude and rate); (2) inner, cell culture reference (as opposed to bare electrodes referencing in classical EIS) is achieved via an optimized pulsatile illumination protocol; (3) exposure to bioactive compounds reproducibly alters the characteristic (reference) cell dynamics upon illumination promoting high sensitivity and rapid cellular response of bio-sensing relevance; (4) the proposed biosensing platform is able to discriminate between compounds with different cell-interaction mechanisms.

### 5.2. Combined Electro-Optical Platforms

The combination of electrical and optical (e.g., surface plasmon resonance—SPR assay and fluorescence microscopy) was demonstrated effective for functional and molecular characterization [109] of subtle bioeffects as well as for the development of label-free, cell-based biosensing platforms as demonstrated for β amyloid effect [31]. Interestingly, the complementarity of EIS and SPR provides additional sensing capabilities [32] by differentiating specific and nonspecific binding and offers new perspectives on plasmonic-based EIS (microscopy) assays. 

In support of combined electro-optical measurements (e.g., optical waveguides, plasmonic-based electrochemical impedance spectroscopy or phase), one can use high-speed image acquisition and processing systems based on complementary metal-oxide semiconductor (CMOS) sensors and field-programmable gate array (FPGA) boards. Such a system [101] allows one to derive the sensitive dependence of the refractive index of electro-optical sensors on surface charge density, modulated by an alternating current, AC, electric field applied to the sensor surface and resolve local impedance with high, optical spatial resolution without using microelectrodes. A comprehensive review of CMOS-based, whole-cell (classical) impedance sensing can be found in Hedayatipour’s work [13].

An electrochemical push–pull probe [99] was proved useful for implementing on cell-based platforms additional analytical tools such as scanning electrochemical microscopy while also allowing multimodal alteration of the cell microenvironment. As demonstrated [25] with an electro-analytic assay with integrated controlled microfluidics and optical stimulation, there is a synergic effect of flow conditions and optogenetic (illumination) control on increasing the cellular reactivity to a noxious compound. The combination of optogenetic stimulation and microfluidics is capable of revealing reproducible effects of the cytotoxic compound (cadmium Cd) at a sub-threshold concentration; it also reveals a large enhancement of cell reactivity by (pre)stimulation with light pulses only accessible in flow conditions and possibly with mechanical stimulation as provided by engineered dynamic hydrogel biomaterials.

Another relevant extension, the high spatial resolution measurement of the extracellular pH of individual cells and cell monolayers was demonstrated using a voltammetric, enzymatic pH microsensor. Therefore, it is conceivable that, by adding the light-guiding properties to such a multifunctional probe, the analytic range can be expanded dramatically.

Other challenges and breakthroughs in the development of analytical tools for cell-based biosensing for specific applications can be found in recent review works [3,4,42,110,111], and thus, we limit the presentation. Instead, a special note is devoted to multimodal functional imaging approaches potentially suitable for ‘all-optical’ electrophysiological cell-based assays under current scrutiny [33,79,112]. ‘All-optical’ electrophysiology concerns the derivation of cellular electrical properties (for instance, membrane and action potentials, ion channels characterization) using only optical means.

### 5.3. Multimodal Functional Imaging for Cell-Based Optogenetic Platforms

The relevance of such an approach is supported by recent developments in cardiac optogenetics [24] that define immediate translation based on ‘all-optical’ electrophysiology, including high-throughput screening and cardiotoxicity testing, thus provide a relevant framework for exploring the challenges and opportunities of cell-based optogenetic sensing.

A multimodal functional imaging instrument (characterized by the dual capability of impedance mapping and phase quantitation) was recently advanced [29] to enable high spatial resolution and low temporal noise maps of optical path differences and electrical impedance variations. When applied to semi-transparent, structured coatings (model for indium tin oxide electrodes effective for cellular platforms development), the imaging instrument can distinguish nano-sized, highly similar electrical contrasts. Heterogeneous interfaces corresponding to an indium tin oxide layer exposed by holes with diameters as small as ~550 nm in a titanium dioxide over-layer deposited on glass support could be quantitatively mapped. The electrical modulation during phase imaging of a macro-electrode was demonstrated decisive for retrieving the electrical impedance distributions with submicron spatial resolution and beyond the limitations of electrode-based technologies (surface or scanning ones).

As such, although the analytic tool has only been demonstrated at the proof-of-concept level on live cell analysis, its suitability for the all-optical electrophysiological cell-based assay is potentially major. To support this assertion, we relate to recent works connecting high spatial resolution extracellular pH measurement of adherently growing mammalian cells [100] with cancer cell versus normal cell discrimination and the dynamic assessment of extracellular space acidification, as well as the all-optical high spatial resolution electrochemical biosensing demonstrated using reflected light microscopy and a macroscopic electrode [102]. In the latter case, a spatial resolution of 12 × 12 μm^2^ (down to the single-cell level) was demonstrated without the need for the fabrication of microelectrodes of these dimensions by combining the electrochemical sensing with optical microscopy (e.g., bright field reflected light microscopy).

Furthermore, advanced computational tools and artificial intelligence (AI) protocols currently in development warrant explosive progress in the field of multimodal assays of cellular platforms. For instance, Cheetah, a flexible computational toolkit that simplifies the integration of real-time microscopy analysis with algorithms for cellular control, has been recently introduced [43]. Central to the platform is an image segmentation system based on convolutional neural networks supplemented with functionality to count, characterize and control (bacterial and mammalian) cells’ growth and protein expression over time. Moreover, a combination of quantitative phase imaging and AI was demonstrated [113,114] to provide information about unlabeled live cells with high specificity suitable for detection for instance of neurite dynamics or intracellular mass changes.

## 6. Wide Biosensing Relevance

Time-lapse fast impedance assays and optogenetic periodic stimulation were demonstrated essential for boosting the cell-platform sensitivity when exposing cells to a model exogenous stimulus, in both static and flow conditions. This ability potentiates breakthroughs in sensing based on non-excitable cell models as well by providing periodic cell membrane potential changes whose recovery to resting values is dependent on cell state and impending bioeffects.

The possibility to apply periodic signals on cells as used in the proposed platform is all the more relevant since pulsing cellular dynamics in genetic circuits have been shown to provide critical capabilities to cells in modulating stress response, signaling and development [115] or initiating patient-specific drug testing when human Pluripotent Stem Cells derived cardiomyocytes were combined with optogenetic pacing [34].

Notable, these fast time-lapse impedance assays are capable of revealing discrete cellular responses associated with opto-chemogenetic biosensing assays: cell micromotion, extension and contraction of lamellipodia or blebbing and exo- and endocytosis [116] effects. The relevance of this aspect is outstanding moreover since it has been demonstrated that all living organisms oscillate at a nanometric scale and that these oscillations stop as soon as the organisms die. Given the ease of analysis and integration of the novel optogenetic noninvasive electro-optical analytical platform, it is conceivably better fitted to assess nanometric scale oscillations than the micro-fabricated cantilever sensors [117] recently proposed as a tool to assess the effect of chemicals on yeast, neurons and cancer cells and for rapid characterization of microorganism susceptibility to pharmaceutical agents.

Moreover, the optogenetic control of G protein-coupled receptors, which are the target of one-third of all marketed drugs [75], has high relevance for the development of bioassays for preclinical testing and drug development.

The advanced optogenetic cell-based approaches and related biomaterials have a wider biosensing relevance:

(a) Design of novel multifunctionality biosensing probes to allow assessment of stimuli induced, normal or pathological aggregation processes. Many proteins undergo aggregation in vitro and in vivo and, as for amyloid type aggregation, this process is involved in the pathology of many degenerative diseases (e.g., amyloid β42 in Alzheimer’s disease or deposits of amyloid lysozyme fibers on the kidney that are characteristic in patients suffering from familial amyloidosis). It is thus of enormous biosensing/biomedical relevance. Indeed, using a label-free platform [31] integrating improved SPR and impedance assays with cell cultures, we showed continuous, quantitative monitoring of cell monolayer under Amyloidβ42 exposure capable of providing a new perspective on the dynamic processes at various levels within an in vitro cellular system. Kaur. et al. [118] demonstrated the use of optogenetic Amyloidβ to monitor in vivo protein aggregation while fluorescent optogenetic Amyloid-beta was shown to enable discrimination between metabolic and physical damages in neurodegeneration [119] as well in vivo settings.
(b) Development of cell-free systems, as part of the synthetic biology field, to become a critical platform in biological studies [120]. The optogenetic tool has been widely proven as an ideal control switch for protein synthesis due to its nontoxicity and excellent time–space conversion. Zhang et al. [120] used a blue light-regulated two-component system to control cell-free protein synthesis and achieve two-way control: a five-fold dynamic protein expression by blue light repression and three-fold dynamic expression by blue light activation. The cell-free blue light-sensing system was used to perform imaging, light-controlled antibody synthesis and light-triggered artificial cell assembly as a proof of principle expansion of optogenetics tools applications in cell-free synthetic biology.
(c) Optical manipulation of protein subcellular localization in cells [35] to be implemented as a way to calibrate new multimodal microscopy [29] tools.
(d) Optogenetic-inspired tools (optogels) to construct light-responsive extracellular matrix (ECM) mimetic hydrogels better mimicking natural ECM [39] and having light adjustable mechanical properties [38]. Optogels have immediate use in dissecting the cellular response to acute mechanical inputs and are suitable extensions towards 3D cellular biosensing platforms.

Moreover, the advanced optogenetic based biosensing and related biomaterials could fuel applications in transversal, emerging domains:

(1) Design and engineer synthetic genetically encoded functional nucleic acids FNAs nanostructures and nanodevices [89], extending the traditional biological roles of nucleic acids as catalytic enzymes, intracellular regulatory molecules and carriers of genetic information towards directing the assembly and functionality of materials at the nanoscale. Versatile FNAs-based, light-controlled nanodevices are expected to be broadly used in the near future to probe and program cells and other biological systems (e.g., regulating and compartmentalizing cellular gene expression, imaging, logic operation).
(2) Design of reporter synthetic cells for environmental, nanotechnology, nanomedicine applications. Functionality gains are widespread: from adding targeted mobility of bio-particles [121] and biohybrid swimmers [122] to designer control of optogenetic-enabled biohybrid cellular sentinels [44,45,46,123]. Optogenetics is poised to decode the minimum instruction set required to direct cell behaviors.
(3) Metabolic cybergenetics [47], i.e., use computer interfaces to enable feedback controls over biological processes and engineered metabolic pathways in real-time.
(4) Novel strategies for designing effective and intelligent drug carriers, novel integrated platforms for targeted drug delivery (e.g., photo-responsive polymersomes for drug delivery [48]).

## 7. Conclusions

The ability to stimulate mammalian cells with light, brought along by optogenetic control, has significantly broadened our understanding of electrically excitable tissues and has paved the way towards novel cellular control tools with relevance for various biomedical applications. More importantly, it has led to a wide variety of optogenetic tools that allow control of cell fate decisions such as proliferation and differentiation as well as modulation of cellular receptors and signaling cascades or delivery of stimuli (of therapeutic relevance) in a spatiotemporally coordinated manner down to the single-cell/organelle resolution whose use in the field of biosensing is often overseen. Aware of the challenges of cell-based biosensors [3] and the potential impact of selected optogenetics tools for bioanalysis, we reviewed the recent literature in the field of (bio)engineered cellular materials and analytic platforms of relevance for the development of engineered cell-based biosensing.

Indeed, optogenetic modified cells offer exciting sensing options and are best represented at the crossroad between smart materials and analytics and cell biology and engineering. Their respective fields show sustained development and provide ready-to-use components. Offering the quantitative means to control cellular phenotypes, integrated multiparametric analysis platforms are envisaged to enable (i) unprecedented spatial and temporal control for cell-based assays, (ii) regulation of intracellular signaling in a combined light- and chemical-dependent manner, (iii) visualization of the spatiotemporal dynamics of cell signaling, (iv) achieving spatiotemporal information about metabolites [124] and regulators (including enzymes) [125] in living cells and in vivo, (v) design of new calibration tools and (vi) new powerful biosensing platforms.

The key advantages of proof-of-concept optogenetically modified, cell-based biosensors [25,26] concern both significantly faster (minutes instead of hours) and higher sensitivity detection of low concentrations of bioactive/toxic analytes (below the threshold concentrations in classical cellular sensors [10]) as well as improved standardization for multiparametric analytics. Given the ease of analysis and integration of the novel optogenetic noninvasive electro-optical analytical platform, the development of more sensitive tools to assess the effect of chemicals and for rapid characterization of susceptibility to pharmaceutical agents is foreseeable in the near future as well as the fulfillment of the heralded promise of cellular biosensing platforms of providing robust and reliable analytic and even theranostic tools.

In view of the importance of optogenetic approaches in these many fields and their use as enabling tools in advanced biosensing, it is conceivable that many ingenious optogenetic sensors and materials will be developed in the future with applications far beyond the current ‘canonic’ biosensing vision.

## Figures and Tables

**Table 1:** Potential applicative fields of optogenetics-powered cell-based biosensing and cellular reporters.

Field	Status	Reference
PON stressor’s bioimpact analysis	Demonstrated and validated proof of concept for an environmental toxicant	[25,26]
Assessment of disease phenotypes and pharmacological modulation	Demonstrated proof of concepts mostly in cardiac research	[33,34]
Design of novel multifunctionality biosensing probes for stimuli induced, normal or pathological aggregation processes	Demonstrated for a fibrillar protein of biomedical relevance	[35]
Reference platforms development	Multiwell Light-Induction Platform	[36]
Design of better cell matrices and 3D constructs for bioanalysis	3D tissue mimic reported; bioanalysis not demonstrated	[37]
	Demonstrated for extracellular matrices	[38]
	Demonstrated for hydrogels	[39,40]
Ingestible/implantable bioelectronic biosensing devices	Potential	[41,42,43]
Theranostics (sense and respond)-oriented cell sentinels	Potential-relevant (connected) progress	[44,45,46,47]
Synthetic cells	Emergent	[48]

**Table 2:** Optogenetic reporter cells relevant for cell-based biosensing platforms.

Target Cells	Optogenetic Molecule	Application	Reference
hIPSC-derived excitatory neurons	CheRiff—voltage actuator QuasAr—voltage reporter	All-Optical Electrophysiology for measurement of intrinsic excitability	[33]
hIPSC-derived cardiomyocytes	ChR2—channel	Frequency-dependent drug screening, high content cardiac toxicity screening or personalized medicine for inherited cardiac channelopathies	[34]
Human fibroblasts	(HE)bacteriorhodopsin—a pump	Optogenetic Modulation and Reprogramming on fullerene C60 nanosheets	[49]
Human embryonic kidney cells HEK * 293 FLPN—2D monolayers	ChR2 alone or with K+ channel	Optogenetic pacing combined with fast electrical impedance assay for quantification of Cd and ouabain	[25,26]
HEK 293 T	ChR2	‘tandem-cell-unit’ method of optogenetic stimulation	[54]
	Cryptochrome 2 (Cry2, fused to mCherry (Cry2-mCh)	Tunable control of protein oligomerization and control of intracellular signaling cascades	[55]
Mouse embryonic stem cells in rod and toroidal fibrin scaffolds	ChR2H134R	Untethered and geometrically stable, functional optogenetic Neural tissue mimics	[37]
Mouse Embryonic stem cells	Melanopsin	Cardiomyocytes (embryoid bodies) with Optogenetic activation of Gq signaling	[56]
Neurons	Various opto tools	Ultra light-sensitive and fast neuronal activation	[57,58]
	Ca^2+^-permeable channelrhodopsin CatCh		[59]
	KillerRed	Permanent inactivation of selected neurons—in Caenorhabditis elegans	[60]
Cardiomyocytes	ChETA_TC_ /R-GECO	Noninvasive phenotyping Drug testing	[41]
Beta cells	ChETA_TC_ /R-GECO	Noninvasive phenotyping	[41]
Plant cells (mesophyll)	ChR2—channel actuator	Optogenetics stimulation combined with voltage-sensing microelectrodes for assessment of stress-associated physiological responses	[50]
Yeasts	Various optogenetic switches (light-controlled on–off gene expression systems)	Optogenetic control of metabolic pathways, heterologous protein production and flocculation	[51] (review)
Yeasts	GPCR-based biosensors Optogenetic switches	Directing protein assembly and controlling metabolic fluxes	[52] (review)
Bacteria *Escherichia* *coli*	Light-switchable (red/far-red and green/red) photo-reversible, two-component signal transduction systems	Hybrid oscillators for detection of sub-inhibitory antibiotic concentrations-generated cell behavior	[53]

* HEK cells are a typical cellular model for optogenetics; (HE)—high efficiency;

**Table 3:** Optogenetic toolbox relevant for biosensing.

Optogenetic Toolbox Component	Property	Wavelength	Effect	Reference
**Actuators**	Opsins	470/570	Membrane polarisation	[23,56,59,65]
Channelrhodopsin Ch	Na^+^ channel	470 nm	Membrane depolarization via internal surface potential shift to positive values	[23]
Calcium translocating channelrhodopsin CatCh	Ca^2+^-permeable channelrhodopsin	473 nm	Membrane depolarization via internal surface potential shift to positive values	[59]
Halorhodopsin HR	Cl^−^ ion pump	570–590 nm	Membrane hyperpolarization	[65]
Archaerhodopsin-3 Arch	Cl^−^ ion pump	570 nm	Membrane hyperpolarization	[23]
Melanopsin	Activation of Gq	480 nm	Membrane depolarization through GPCR signaling cascade	[56]
OptoXR Opsin/G protein- coupled receptor chimeric molecules	Activation of Gq	470 nm	Merge an extracellular light sensitive part and an intracellular G protein-coupled receptor to initiate a signaling cascade upon activation	[56]
**Actuators**	Non-opsin-based	various	various	[66,67]
Light-oxygen-voltage-sensing domain (LOV) cryptochrome (CRY2) phytochrome (PhyB and BphP)	various	various	various	[66]
fluorescent protein (FP)-based photosensitive domains (Dronpa and PhoCl)	various	various	various	[67]
Light activated enzyme—killer red	various	various	ROS generation	[66]
**Sensors**	various	various	various	[41,68,69]
Optogenetic Ca^2+^ indicator probes (e.g., red calcium indicator protein, R-GECO)	various	various	Ca^2+^ indicator probes of G protein-coupled receptors activation	[41]
	various	various	Ca^2+^ indicator probes of functional cell–cell communication	[68]
Traceable intracellular binding molecules—Intrabodies	various	various	Antibody fragments of heavy-chain only antibodies of camelids (nanobodies) to visualize bioactive antigens	[69]
**Opto-switches**	various	Various excitation/reversion wavelengths	The transfer of biochemical information from sensor domain to the actuator domain is mediated by conformational changes and aggregation processes that are able to be switched on and off	[27] and https://www.optobase.org/switches [27]
UV receptors	receptors	300nm/dark	Heterodimerization/homodimerization, dissociation	[27]
Cyanobacteriochromes CcaS/CcaR	photoreceptor	535/670	Gene expression	[27]
BLUF domains bPAC (BlaC)	-	450/dark	cAMP production	[27]
LOV (light, oxygen, voltage) domains	various	various	various	[27,66,67]
Cryptochromes	various	various	various	[27,66]
Fluorescent proteins (Dronpa, PhoCl, PYP)	various	various	/photocleavage/	[27,66,67]
Phytochromes	PhyB	660/740	heterodimerization	[66,67]

BLUF—sensors of blue-light using flavin adenin nucleotide; BphP—bacteriophytochrome Pseudomonas Aeruginosa; CcaS/CcaR cyanobacteriochrome PhoCl—photocleavable protein using violet-light; PhyB—phytochromeB; PYP—photoactive yellow protein; R-GECO—red calcium indicator protein.

## Data Availability

All the data is available within the manuscript.
